# Intrinsic magnetic properties of single-phase Mn_1+x_Ga (0 < x < 1) alloys

**DOI:** 10.1038/srep17086

**Published:** 2015-11-24

**Authors:** Q. M. Lu, M. Yue, H. G. Zhang, M. L. Wang, F. Yu, Q. Z. Huang, D. H. Ryan, Z. Altounian

**Affiliations:** 1 College of Materials Science and Engineering, Beijing University of Technology, Beijing, 100124, China; 2 NIST Center for Neutron Research, 100 Bureau Drive, Mailstop 6102 Gaithersburg MD, 20899-6102, USA; 3 Center for the Physics of Materials and Department of Physics, McGill University, 3600 University Street, Montreal, Quebec, H3A 2T8 Canada

## Abstract

Magnetization measurements have been carried out on a series of carefully prepared single-phase Mn_1 + x_Ga (0 < x < 1) alloys. The saturation magnetization M_s_, measured at 5 K, has a value of 92.0 emu/g for x = 0.15. This is the highest value reported in these alloys and is close to the calculated value of 116 emu/g for the stoichiometric compound (x = 0). M_s_ decreases gradually with x and has a value of 60.7 emu/g for x = 0.86. This behavior is consistent with the extra Mn atoms occupying Ga sites and coupling antiferromagnetically with the rest of the Mn atoms. The intrinsic magnetic properties of the Mn-Ga alloys indicate their great potential as novel, rare-earth free permanent magnetic materials.

Ferromagnetic tetragonal P4/mmm (also known as L1_0_) MnGa containing neither rare earth nor noble metal atoms is a promising candidate for novel permanent magnetic materials due to its high Curie temperature (T_C_) and strong magnetic anisotropy field (H_a_). MnGa, in theory, possesses a saturation magnetization (M_s_) of 116 emu/g^1^,^2^, which could potentially yield a maximum energy product, (BH)_max_, of 28 MGOe. Unfortunately, stoichiometric MnGa cannot be manufactured due to preparation difficulties^3^,^4^. To date, several groups have reported structural and magnetic properties of Mn_1 + x_Ga alloys from ball milled powders^3^, melt-spun ribbons^4^, and thin films^5^,^6^,^7^. However, the reported values of M_s_ for the milled powders and thin films are about 50% lower than the predicted value of 116 emu/g. The highest value of 85 emu/g was reported for Mn_1.2_Ga melt-spun ribbons by Huh *et al.*^4^.

In tetragonal Mn_1 + x_Ga (0 < x < 1) alloys, belonging to either the ordered P4/mmm (L1_0_) or the disordered I4/mmm (D0_22_) structures, the magnetization of the alloys decreases with increasing Mn content, x. This tendency has been attributed to antiferromagnetic coupling between the excess Mn atoms on the Ga (2b) site and their neighbouring Mn atoms on the 4d site^8^. To explain this effect, several structural, crystalline and magnetic, models have been proposed. Based on the ordered structure, Mizukami *et al.*^8^ have suggested that the extra Mn atoms can randomly replace Ga atoms at the 2a or 2b sites and couple antiferromagnetically with the Mn atoms at the 4d site. For the disordered structure, on the other hand, Winterlik *et al.*^9^ proposed two models: one involving Mn vacancies at the 2b site, and the other the Mn vacancies at both the 2b and 4d sites in the unit cell^9^. However, the exact atomic arrangement in these tetragonal Mn_1 + x_Ga alloys remains unknown.

To introduce a practical hard magnet, three basic steps must be followed. First and foremost is the determination of the intrinsic magnetic properties (saturation magnetization, anisotropy field and Curie temperature) of the material. If the results are encouraging, the next step is to determine the magnetic structure. This step is important in improving the magnetic properties through elemental substitutions. The last step is the enhancement of the extrinsic magnetic properties, such as remanence and coercivity, through control of the microstructure. A look at the Mn-Ga phase diagram shows that this is one of the most complex binary phase diagrams. To avoid complexities and the effects of the microstructure on the magnetic properties, as seen in thin films and nanograin samples^10^,^11^,^12^, we need to prepare good quality single-phase samples (see *Methods*). In the present study, we report on the intrinsic magnetic properties of single-phase Mn_1 + x_Ga (0 < x < 1) alloys.

## Results and Discussion

**Figure 1:**
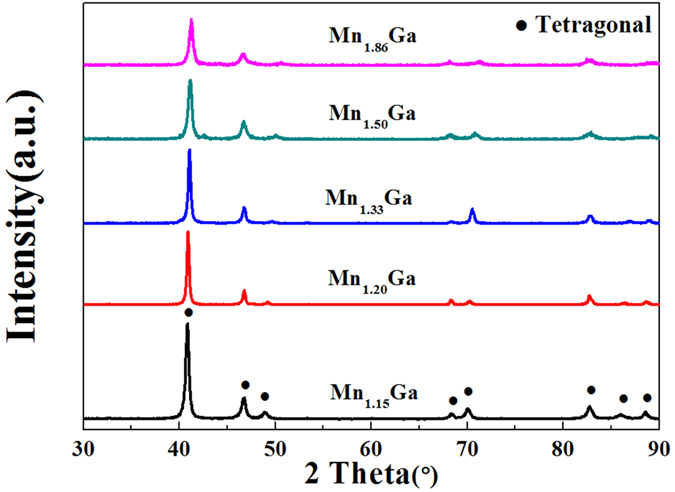
X-ray diffraction patterns of Mn_1 + x_Ga.

The X-ray diffraction results, as shown in Fig. 1, confirm that all the alloys are single phase, and can be fitted to the tetragonal structure. The lattice parameters as well as the volume of the unit cells for all the alloys are displayed in Table 1. The reduction in Mn content is associated with a slight contraction in *a* and an expansion along *c*, leading to a small overall volume increase of the unit cell.

**Figure 2:**
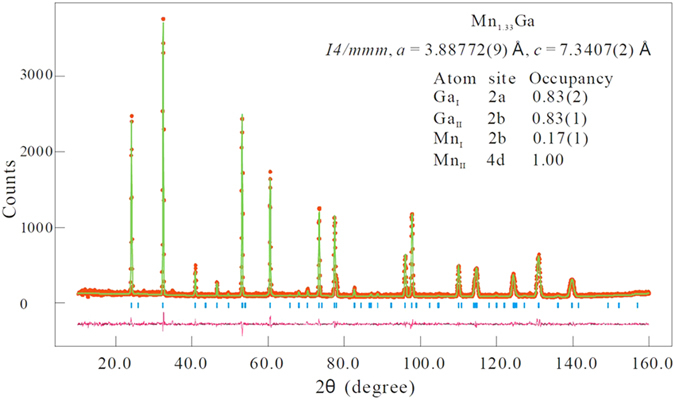
Room temperature neutron powder diffraction pattern for Mn_1.33_Ga. The red circles denote the experimental observations and the green line is one of the model calculations with the occupancies as shown in the inset. The vertical blue lines are the Bragg positions and the difference between the measurement and the fit is shown by the magenta line the bottom.

**Figure 3:**
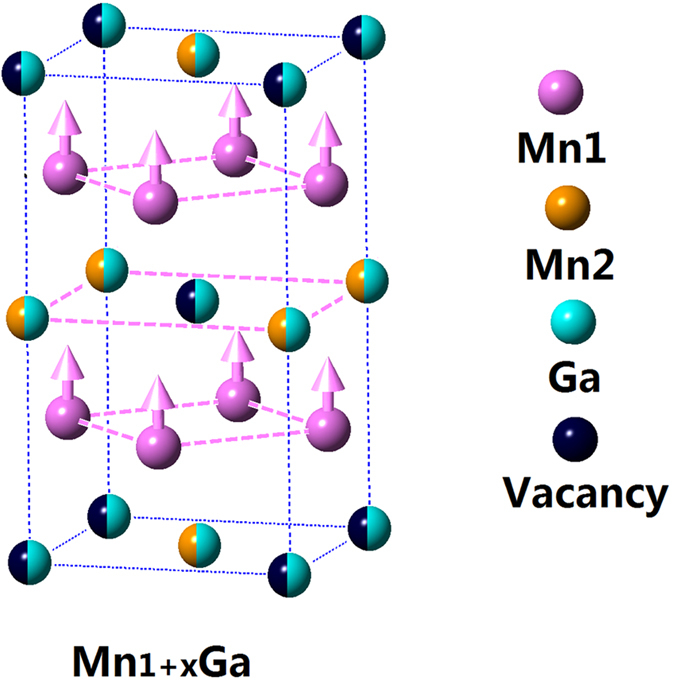
The disordered tetragonal structure (I4/mmm) for Mn_1 + x_Ga.

The neutron diffraction pattern for Mn_1.33_Ga is shown in Fig. 2. We only show the neutron diffraction pattern to confirm the single-phase nature of the alloy. The refinement results will not be given here as we could not obtain a unique fit to the data. This was due to the number of parameters involved in the fitting. These include: the number and location of possible Ga vacancies for x > 0, possible disorder among the Mn/Ga atoms at different sites, the magnitude and direction of the magnetic moments of Mn on the two sites. Fits to the neutron results could be obtained with the same χ^2^ value but with quite different values for the above noted parameters that were consistent with the nominal composition (x) and the experimental M_s_. For this reason, we cannot make any comment on previously published fitting results on Mn_1 + x_Ga. We are in the process of carrying out an extensive neutron diffraction study for all the compositions to obtain a complete description for the structure. The only conclusion we can draw from our diffraction (XRD and NPD) experiments is that the *most likely* structure for all the alloys is the disordered tetragonal structure with space group I4/mmm as shown in Fig. 3. For MnGa (x = 0), the *c*-axis is half of that shown in the figure. In the ordered structure, P4/mmm, there are no vacancies and no Ga/Mn substitutions while for the disordered structure, I4/mmm, Mn/Ga substitutions can occur as well as the possibility of vacancies at the Ga 2a sites.

**Figure 4:**
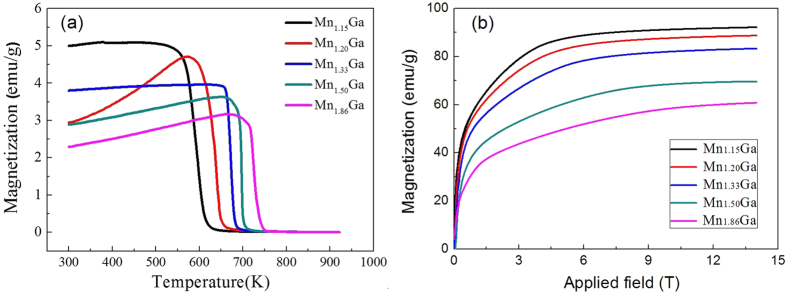
Magnetization as a function of temperature (a) and applied magnetic field (b) for Mn_1 + x_Ga alloys.

Figure 4(a) shows the magnetization as a function of temperature for some of the alloys under an applied field of 0.01 T, and the corresponding field dependence of the magnetization is shown in Fig. 4(b). The values for the saturation magnetization, M_s_ (in both emu/g and, in parentheses, Bohr magnetons per formula unit), the anisotropy field, H_A_, as well as the Curie temperature, T_c_, are listed in Table 2.

One can see that for Mn_1.15_Ga the saturation magnetization (M_s_) has a value of 92.0 emu/g, which is amongst the highest reported M_s_ of rare earth free permanent magnetic materials.

Before analyzing the results we decided that it would be prudent to recalculate the magnetic moment per unit cell of MnGa using the most accurate DFT calculation with the FLAPW (full potential linearized plane-wave) code. Our result is in very good agreement with that of references^1^,^2^.

The influence of Mn content on the intrinsic magnetic properties of Mn_1 + x_Ga alloys is clearly seen in Table 2. As the Mn content decreases from 1.86 to 1.15, the M_s_ of the alloys increases rapidly from 60.7 emu/g to 92.0 emu/g. This change in M_s_ is consistent with the model proposed in ref. 8. On the other hand, H_a_ and T_C_ of the alloys decrease monotonically with the decrease of Mn content. Even with this decrease, H_a_ and T_C_ of the Mn_1.15_Ga alloy are 66 kOe and 595 K, respectively, which are comparable with those of Nd_2_Fe_14_B. Therefore, the overall intrinsic magnetic properties (M_s_, H_a_, and T_C_) of the Mn_1 + x_Ga alloys (especially for alloys with x ≤ 0.33) indicate their great potential as novel permanent magnetic materials.

**Figure 5:**
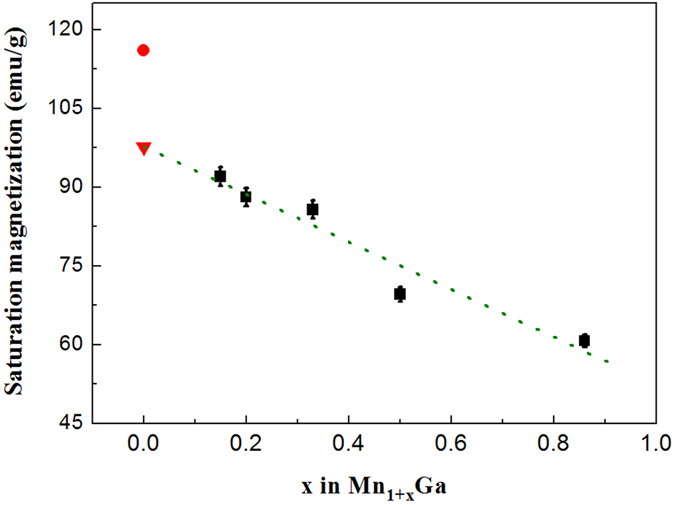
Composition dependence of saturation magnetization at 5 K (black squares), of Mn_1 + x_Ga alloys. The red circle denotes the calculated value^1^, and the green dotted line is a linear fit to our data.

Figure 5 shows the composition dependence of the saturation magnetization, measured at 5 K, of the Mn_1 + x_Ga alloys. The solid red square denotes the calculated value for MnGa (x = 0). A linear extrapolation of the data, green dotted line, predicts a value of M_s_ = 98 emu/g for MnGa. This is 15% lower than that calculated for the ordered MnGa structure. The difference between the extrapolated and the calculated values is most likely due to the extrapolation from the disordered Mn_1 + x_Ga structures. Nevertheless, even this reduced M_s_ gives a maximum energy product of 20 MGOe for MnGa.

To achieve a high (BH)_max_ in MnGa alloys, however, major experimental efforts are necessary. For example, magnetic hardening should be accomplished in advance since the high coercivity is crucial for permanent magnets. Equally important is to induce a strong crystallographic texture to provide a high remanence ratio (M_r_/M_s_) leading to a high (BH)_max_.

## Conclusion

In summary, structure and intrinsic magnetic properties were studied for a series of single-phase Mn_1 + x_Ga (0 < x < 1) alloys. The net magnetic moment in the Mn_1 + x_Ga system increases monotonically with decreasing Mn content. This behavior is consistent with extra Mn atoms occupying Ga sites and coupling antiferromagnetically with the rest of the Mn atoms. We report the highest value of 92 emu/g for the x = 0.15 alloy. Mn_1 + x_Ga alloys (x ≤ 0.33) exhibit excellent intrinsic magnetic properties. An extrapolated value of 20 MGOe for (BH)_max_ is predicted for stoichiometric disordered MnGa alloy, demonstrating the great potential of Mn_1 + x_Ga alloys as novel permanent magnetic materials.

## Methods

A series of tetragonal Mn_1 + x_Ga (x = 0.15, 0.20, 0.33, 0.50, and 0.86) alloys were prepared by induction melting high purity gallium (99.9 wt.%) and manganese (99.5%) in an argon atmosphere. To compensate for evaporation losses during melting, an extra 3 wt.% Mn was added to the alloys. The as-melted ingots were then annealed in a tubular vacuum furnace at temperatures, T_a_, ranging from 700 K to 900 K for annealing times, t_a_, extending from one day to up to one week. The ingots were subsequently quenched into ice water. The annealing step is crucial for obtaining single-phase alloys. Due to the complexity of the Mn-Ga binary phase diagram, the annealing temperature rangeis quite narrow and the optimum choice depends critically on the composition of the alloy. The annealing temperatures were 740 K, 825 K and 870K for x = 0.15, 0.20 and 0.33, respectively, and 885K for the alloys with higher Mn content. While the annealing temperature was critically important, the annealing time did not appear to play a major role and we did not observe any significant differences between samples annealed for 2 and 7 days. The crystal structures were determined by x-ray diffraction with Cu-K_α_ radiation. For one composition, x = 0.33, the structure was investigated using neutron powder diffraction (NPD) datacollected on the BT1 high-resolution powder neutron diffractometer located at the NIST Center for Neutron Research with monochromatic neutrons of wavelength 1.5403 Å produced by a Cu (311) monochromator. Rietveld refinement was performed using the GSAS/EXPGUI program^13^. Magnetic properties were measured using a Quantum Design physical properties measurement system (PPMS) magnetometer with a maximum magnetic field of 14 T. Temperature dependence of the magnetization was obtained from a vibrating sample magnetometer (VSM) using a field of 0.01T. From the initial magnetization and corresponding differential susceptibility curves, the values for the anisotropy field, H_A_, were obtained.

## Additional Information

**How to cite this article**: Lu, Q.M. *et al.* Intrinsic magnetic properties of single-phase Mn_1+x_Ga (0 < x < 1) alloys. *Sci. Rep.* **5**, 17086; doi: 10.1038/srep17086 (2015).

## Figures and Tables

**Table 1:** Lattice constants and cell volumes of Mn_1 + x_Ga alloys.

Composition	Lattice constants		
Mn_1 + x_Ga	*a*(Å)	*c*(Å)	*V*(Å^3^)
Mn_1.15_Ga	3.8796	7.4280	111.80
Mn_1.20_Ga	3.8812	7.3878	111.29
Mn_1.33_Ga	3.8877	7.3407	110.95
Mn_1.50_Ga	3.8934	7.2918	110.53
Mn_1.86_Ga	3.8992	7.2221	109.80

**Table 2:** Intrinsic magnetic properties of Mn_1 + x_Ga alloys (M_s_ and H_a_ are measured at 5 K).

Sample	Intrinsic magnetic properties		
Mn_1 + x_Ga	M_s-14T_ (emu/g)/(μ_B_/f.u.)	H_a_ (kOe)	T_C_ (K)
Mn_1.15_Ga	92.0/2.19	66	595
Mn_1.20_Ga	88.1/2.14	69	640
Mn_1.33_Ga	85.7/2.19	75	673
Mn_1.50_Ga	69.6/1.90	80	697
Mn_1.86_Ga	60.7/1.87	94	738
